# Determinants of Increased Serum Calprotectin in Patients with Type 2 Diabetes Mellitus

**DOI:** 10.3390/ijms21218075

**Published:** 2020-10-29

**Authors:** Milou M. Oosterwijk, Stephan J.L. Bakker, Tom Nilsen, Gerjan Navis, Gozewijn D. Laverman

**Affiliations:** 1 Department of Internal Medicine/Nephrology, Ziekenhuis Groep Twente, Zilvermeeuw 1, 7609 PP Almelo, The Netherlands; mi.oosterwijk@zgt.nl; 2 Department of Internal Medicine, Division of Nephrology, University of Groningen, University Medical Center Groningen, Hanzeplein 1, 9713 GZ Groningen, The Netherlands; s.j.l.bakker@umcg.nl (S.J.L.B.); g.j.navis@umcg.nl (G.N.); 3 Gentian Diagnostics AS, Bjornasveien 5, 1596 Moss, Norway; tom.nilsen@Gentian.no

**Keywords:** calprotectin, type 2 diabetes mellitus, cardiovascular diseases, lifestyle, smoking, biomarker, endovascular inflammation

## Abstract

Circulating calprotectin is a potential biomarker for endovascular inflammation in type 2 diabetes mellitus (T2DM). We investigated the determinants of calprotectin and its relationship with the presence of cardiovascular disease (CVD) in 362 T2DM patients included in the Diabetes and Lifestyle Cohort Twente-1 (DIALECT-1) study. Lifestyle exposures, including nutrition, were determined by validated questionnaires. CVD was defined as coronary artery diseases, strokes, and peripheral artery diseases. Median serum calprotectin levels were 1.04 mg/L [IQR: 0.73–1.46 mg/L] and were higher in women (1.11 mg/L) than men (0.96 mg/L, *p* = 0.007). Current smoking was a major independent determinant of circulating calprotectin, with a 51% higher calprotectin compared to never smoking (*p* < 0.001). Albuminuria (*p* = 0.011), former smoking (*p* = 0.023), and intake of mono- and disaccharides (*p* = 0.005) also contributed independently to circulating calprotectin. Each incremental increase in calprotectin level was associated with 1.36-times higher odds for CVD (95% CI 1.04–1.77, *p* = 0.026). In the current study, calprotectin was the only inflammatory parameter significantly associated with CVD. The strong association of circulating calprotectin with smoking, a well-known direct cause of vascular inflammation, and also with CVD, stresses the urge for further research to define its role as a biomarker in T2DM.

## 1. Introduction

Calprotectin, also known as S100A8/A9 or MRP8/14, is a protein that is secreted by leukocytes. The expression of calprotectin is upregulated in response to inflammation, and elevated serum concentrations of calprotectin have been reported in multiple chronic inflammatory conditions, including rheumatoid arthritis and inflammatory bowel diseases [1,2,3,4]. Recently, calprotectin was found to be increased in subjects with insulin resistance and obesity [5,6,7].

In line with this, higher serum levels of calprotectin have been found in persons with type 2 diabetes mellitus (T2DM) compared to healthy individuals [8]. Serum calprotectin has also been shown to be associated with complications of diabetes, including cardiovascular disease (CVD) [9,10].

Despite accumulating evidence concerning serum calprotectin and its association with insulin resistance and CVD, little is known about the potential influence of lifestyle factors such as smoking, physical activity and nutrition, on circulating concentrations of calprotectin. Particularly, ingestion of modern Western food is considered to be pro-inflammatory and to be associated with hyperinsulinemia and insulin resistance [11].

In this study we aimed to assess determinants associated with an increased serum calprotectin in patients with T2DM. In addition, we investigate the potential role of calprotectin as a biomarker in the development of CVD, compared to already established markers of inflammation.

## 2. Results

### 2.1. Baseline Characteristics

The study population (*n* = 362) consisted of 57% male participants, mean age was 63 ± 9 years, and mean BMI was 32.7 ± 5.8 kg/m^2^ (Table 1). The median serum calprotectin level was 1.04 mg/L [IQR: 0.73–1.46 mg/L], with significantly higher levels in women (1.11 mg/L [IQR 0.83–1.53]) than in men (0.96 mg/L [IQR 0.65–1.42], *p* = 0.007). Current smoking (*r* = 0.266, *p* < 0.001), former smoking (*r* = 0.135, *p* = 0.022), albuminuria (*r* = 0.135, *p* = 0.011) and intake of mono- and disaccharides (*r* = 0.127, *p* = 0.015) were positively associated with serum calprotectin levels.

### 2.2. Inflammatory Parameters

**Figure 1:**
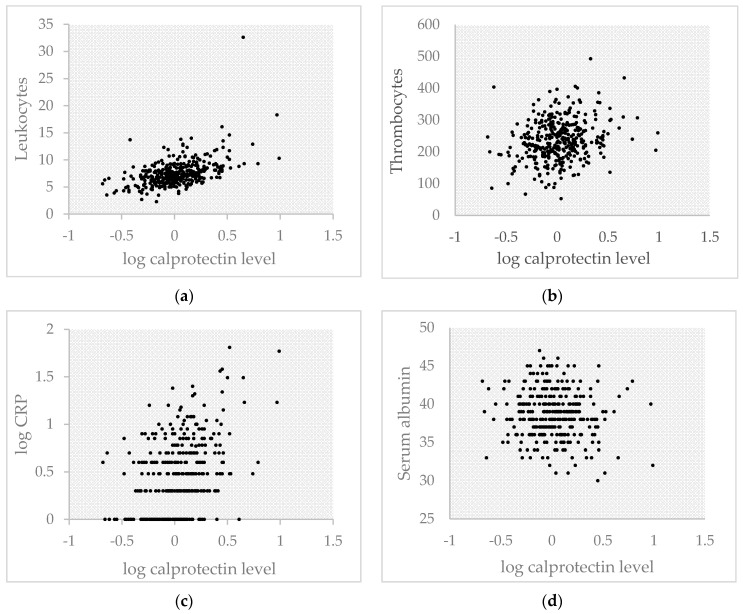
Scatterplots between log calprotectin and various inflammation parameters: (**a**) Scatterplot between log calprotectin and leukocytes (10^9^/L); (**b**) Scatterplot between log calprotectin and thrombocytes (10^9^/L); (**c**) Scatterplot between log calprotectin and log CRP (mg/L); (**d**) Scatterplot between log calprotectin and serum albumin (g/L).

We investigated the potential correlation with established inflammatory markers separately. Scatterplots for serum calprotectin versus these markers are shown in Figure 1. Moderate positive correlations with serum calprotectin were found for leukocytes (Figure 1a, *r* = 0.499, *p* < 0.001) and C-Reactive Protein (CRP) (*r* = 0.419, *p* < 0.001). A weak positive correlation with serum calprotectin was found for thrombocytes (*r* = 0.230, *p* < 0.001), whereas the inverse correlation with serum albumin was not significant (*r* = −0.083, *p* = 0.12).

### 2.3. Independent Determinants of Serum Calprotectin

Plasma calprotectin was found to be independently associated with smoking status, intake of mono- and disaccharides and albuminuria (Table 2).

Current smoking was the major independent determinant of circulating calprotectin; patients who smoke had a 51% (95% CI 27 to 79) higher log calprotectin value compared to persons that have never smoked (*p* < 0.001) (Table 3). Moreover, albuminuria (18%, 95% CI 4 to 34, *p* = 0.011), former smoking (16%, 95% CI 2 to 33, *p* = 0.023) and intake of mono- and disaccharides (0.2%, 95% CI 0 to 0.5, *p* = 0.005) contributed independently to circulating calprotectin after controlling for the effects of age and sex.

### 2.4. Serum Calprotectin Related to CVD

At baseline, *n* = 133 patients suffer from CVD (37%). In the fully adjusted model, each incremental increase in calprotectin level was significantly associated with 1.36-times higher odds for CVD (95% CI 1.04–1.77, *p* = 0.026) (Table 4).

**Figure 2:**
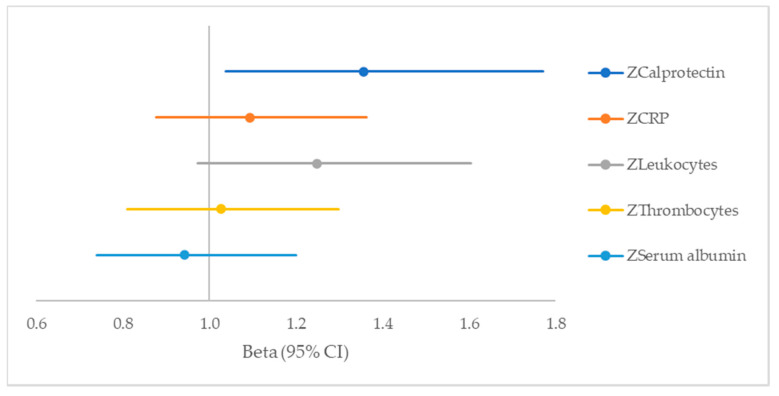
Forest plot with serum inflammation parameters as predictors for CVD in 362 patients with T2DM in the DIALECT-1 population.

Compared with other inflammatory parameters, only calprotectin is significantly associated with CVD (Figure 2).

### 2.5. Sensitivity Analyses

We performed sensitivity analyses in which we repeated analysis after addition of polysaccharides, fatty acids and dietary protein as confounders in the fully adjusted model. The results of these analyses remained materially unchanged compared with the primary analyses. Addition of HbA1c (mmol/mol), insulin use (*n*, %) and diabetes duration (years) to the multivariate logistic regression model for CVD did not materially change the standardized Beta (95% CI) for the association of the Z-score of log calprotectin with CVD (1.356 (1.037–1.773), *p* = 0.026).

## 3. Discussion

In this cross-sectional study among 362 patients with T2DM, we investigated the potential influence of lifestyle factors on circulating concentrations of calprotectin. The major findings in this study were (1) serum calprotectin levels are log-linearly correlated with sex, smoking status, intake of mono- and disaccharides and albuminuria, (2) serum calprotectin levels are associated with CVD, and (3) serum calprotectin levels have a strong predictive potential relative to other inflammation parameters.

Median calprotectin levels in our study population were higher than median calprotectin level in the Dutch general population (1.04 mg/mL versus. 0.50 mg/L) [5]. Our findings are in line with previous studies, which have shown higher serum calprotectin levels in patients with T2DM compared to patients without T2DM. Furthermore, the same holds true for serum calprotectin concentrations in T2DM patients with CVD, which are significantly higher compared to diabetic patients without CVD [10]. Whereas BMI has previously been demonstrated to be positively associated with higher serum calprotectin levels in a lower range, this was not the case in our population with a mean BMI of 32.7 kg/m^2^, suggesting that such a relationship does not exist in a higher range of body mass and/or is lost in patients with established T2DM. However, our study does indicate a relationship between body composition and serum calprotectin as demonstrated by the negative association with muscle mass, and the positive association with body fat percentage (the latter was measured in a subset of *n* = 154 patients), which is consistent with the concept of adipose tissue being actively involved in the pathophysiology of inflammation [12].

Plasma calprotectin was found to be independently associated with smoking status, intake of mono- and disaccharides, and albuminuria. Higher serum calprotectin levels were associated with smoking, a well-known direct cause of endovascular inflammation [13]. Smoking may stimulate the formation of reactive oxygen species (ROS) [14], resulting in oxidative stress and thus significant damage to cell structures, i.e., a key feature in the development of atherosclerosis [15]. Dietary sugar consumption, too, can fuel inflammatory processes in humans [16]. In addition, it appears that diets with lower carbohydrate content and higher contents of fat, fiber and protein may reduce inflammation in patients with T2DM [17]. This pathway is possibly related to glycated proteins and lipids, as a result of exposure to sugars. These advanced glycation end products (AGE’s) trigger inflammatory responses [18,19]. Previous studies suggest that calprotectin binds to receptors for AGE’s [20]. The presence of microalbuminuria was previously shown to be associated with higher calprotectin levels compared to normoalbuminuria, which is confirmed by our results [21]. Elevated levels of inflammatory markers may be the result of pre-existing atherosclerosis in subjects with albuminuria [22].

We found that plasma calprotectin is associated with the presence of CVD in patients with T2DM, corresponding with previous literature [9,10]. Although the design of our study does not allow us to draw conclusions on this, these results, together with the results above, suggest that calprotectin is an indicator of the vascular inflammatory process culminating in CVD. This renders it worthwhile to further investigate the role of calprotectin, first its role in the pathophysiology of inflammation and, second, the potential role as biomarker for vascular inflammation.

After adding CRP, thrombocytes and serum albumin to the fully adjusted model, plasma calprotectin still predicted the presence of CVD, corresponding to the PREVEND findings in which the association between plasma calprotectin and CVD remained present after adjustment for CRP [5]. This suggests that calprotectin as an inflammation marker is complementary to other inflammation parameters, and that calprotectin could be a potential new therapeutic target. After adding leukocytes as inflammation markers to the fully adjusted model, the association between calprotectin and CVD was not significant anymore, potentially explained by the fact that calprotectin is mainly secreted by neutrophils [23,24].

The strength of the DIALECT-1 cohort is that it reflects the real world of patients with T2DM and it was specifically designed to evaluate lifestyle characteristics. Obviously, the cross-sectional nature of the study prevents us from making causal inferences about the role of calprotectin in the pathway of the inflammatory process. Despite the fact that HbA1c, insulin use and/or diabetes duration were not significantly correlated with log calprotectin, and that addition of these variables to the multivariate logistic regression model for CVD did not materially change the standardized Beta (95% CI) for the association of the Z-score of log calprotectin with CVD, the accumulated evidence from prior studies makes it likely that glycosylation is an important contributor to the occurrence of CVD [25]. It cannot be excluded that increased calprotectin is related with level of glycemia management. Diet was assessed by means of a validated extensive 177-item Food Frequency Questionnaire (FFQ) which enabled adequate ranking of individuals for a large number of nutrients. Misclassification may have occurred, because the method is based on self-report, which may have attenuated the observed associations. Furthermore, because of the cross-sectional nature of the study, we cannot exclude the possibility that patients have changed their diet due to health complaints, which may have introduced reverse causation bias.

The unavailability of a matched population of patients without T2DM limits the generalizability of our study results. We are aware of the fact that patients with T2DM have higher serum calprotectin levels compared to the general Dutch population. Previous research suggests that also in the general population further assessment of the role of calprotectin in CVD prevention is warranted, despite the fact that higher serum calprotectin levels were associated with smoking and with the presence of CVD. However, after adjustment for among others smoking status and history of diabetes, higher calprotectin levels were associated with a higher Hazard Ratio for CVD in the general population [5].

The prognostic value of calprotectin in patients with T2DM may have several implications for the development of strategies to prevent micro- and macrovascular complications. However, further studies are required to evaluate its prognostic and diagnostic potential, e.g., investigations to indicate whether calprotectin might aid in the early identification of T2DM people at high risk of developing complications. Furthermore, the therapeutic potential is of interest for assessment and monitoring of disease activity in patients with T2DM, since calprotectin decreases for instance after weight loss [26].

## 4. Materials and Methods

### 4.1. Study Design

We performed cross-sectional analyses using baseline data of the DIAbetes and LifEstyle Cohort Twente-1 (DIALECT-1) study. DIALECT-1 is an observational prospective cohort study performed in the Ziekenhuis Groep Twente (ZGT) Hospital (Almelo and Hengelo, The Netherlands) which investigates the effect of lifestyle and dietary habits on outcomes in patients with T2DM. Patients in DIALECT-1 were included between September 2009 and January 2016. The study was performed according to the guidelines of good clinical practice and the Declaration of Helsinki. Written informed consent was obtained from all subjects prior to participation. The study was approved by the local institutional review boards (METC-registration numbers NL57219.044.16 and 1009.68020) and is registered in the Netherlands Trial Register (NTR trial code 5855).

### 4.2. Patients

The study population consists of T2DM patients aged ≥18 years, treated in the outpatient clinic of the ZGT hospital. Patients on renal replacement therapy or patients with insufficient knowledge of the Dutch language were excluded from participation.

From 450 patients that were included, 11 patients were excluded from the study as their correct diagnosis appeared to be type 1 diabetes mellitus, one patient was excluded because of diabetes development after pancreatitis, two patients were excluded because they had renal replacement therapy prior to baseline measurements and from three patients we excluded the last baseline visit because they participated twice in the study, resulting in a total number of 433 patients eligible in DIALECT-1 [27]. For the current study, we excluded patients with missing serum calprotectin data (*n* = 35), patients with missing albuminuria data (*n* = 17), patients with missing data on dietary intake (*n* = 11), patients with missing urinary sodium excretion data (*n* = 5) and patients whose energy intake exceeded the borders of ± 3SD of the mean energy intake (*n* = 3), leaving 362 patients for analysis.

### 4.3. Dietary Assessment

Nutritional intake was assessed by using a semi-quantitative 177-item Food Frequency Questionnaire (FFQ) [28]. Patients reported food and beverages intakes, including alcohol, over the past month. For each item, the frequency was recorded in times per day, week or month. The number of servings was expressed in natural units (e.g., slice of bread or apple) or household measures (e.g., cup or spoon). Questionnaires were checked for completeness by a trained researcher, and inconsistent answers were verified with the patients. Dietary data were converted into daily nutrient intakes using the Dutch Food Composition Table of 2013 [29]. In August 2019 nutritional data were revised and discrepancies between original data and real data of two food items were captured.

### 4.4. Outcome Measurement

Serum calprotectin levels were measured using Gentian Calprotectin turbidimetric immunoassay (Gentian, Moss, Norway) applied on a Mindray BS-400 analyser (Mindray, Shenzhen, China). Calprotectin concentration in serum is presented in mg/L and reference value in healthy volunteers is <3 mg/L.

### 4.5. Covariates

During the study visit, we collected information relevant to the medical condition and pharmacological treatment. Weight (kg), height (cm), waist- and hip circumference (cm) were measured, and the Body Mass Index (BMI) was calculated (weight divided by squared height). Information about lifestyle exposures, e.g., smoking and physical activity was collected by a self-administered questionnaire. Physical activity was assessed by the previously validated Short Questionnaire to Assess Health enhancing physical activity (SQUASH) questionnaire [30]. An activity score is calculated based on minutes of activity per day multiplied by an intensity factor. From these data, we scored which patients meet the Dutch Healthy Exercise Norm of 30 min moderate intense activity a day for at least 5 days a week [31]. Alcohol consumption was divided in three groups: no alcohol consumption, <1 unit per day and >1 unit per day.

Patients were asked to collect their 24-h urine to obtain the urinary excretion of sodium as objective measure of sodium intake, by multiplying these concentrations with the volume of the 24-h urine collection. Muscle mass was estimated by 24-h urinary creatinine excretion rate (CER). Patients were instructed to store the canister in a dark cool place, preferably in a refrigerator. Routine laboratory tests were performed in non-fasting venous blood, including renal function tests and HbA1c. Blood pressure was measured in a supine position by an automated device (Dinamap^®®^; GE Medical systems, Milwaukee, WI, USA) for fifteen minutes with a one-minute interval. The mean systolic and diastolic blood pressure of the last three measurements was used for further analysis.

Renal function was assessed by the estimated Glomerular Filtration Rate (eGFR), based on the Chronic Kidney Disease Epidemiology Collaboration formula, which is based on serum creatinine levels (umol/L), sex and age. Microalbuminuria was defined as levels of albumin above 30 mg in a 24-h urine collection. Cardiovascular complications included coronary artery diseases (unstable angina pectoris, myocardial infarction, percutaneous coronary intervention, or coronary artery bypass graft), strokes (transient ischemic attack, cerebrovascular accident) and peripheral artery diseases (aneurysms, dilatation of the aorta, atherosclerosis, carotid plaque, carotid endarterectomy). The physician-diagnosed cardiovascular conditions were derived from the medical history and verified during the baseline visit.

### 4.6. Statistical Analysis

All statistical analyses were performed using IBM SPSS for Windows (version 23.0., IBM Corp.: Armonk, NY, USA). Normally distributed data are presented as mean (standard deviation), skewed variables as median [interquartile range] and dichotomous variables as number (percentage). A two-tailed *p*-value less than 0.05 was considered statistically significant.

Serum calprotectin was a skewed variable, and log transformed to achieve approximately normal distribution (log calprotectin level). Sex-adjusted standardized linear regression coefficients were estimated to assess the cross-sectional correlations between various determinants and calprotectin levels. Potential determinants were based on previous literature regarding inflammation. Strength of correlation coefficients was graded according to generally accepted recommendations [32].

All univariate variables with a *p*-value below 0.20 were included in a forward linear regression model with log calprotectin level as continuous outcome. Variables that remain significant were tested in each model, until full adjustment of the model. Model 1 was adjusted for age and sex, model 2 was additionally adjusted for smoking status (current, former, never) and model 3 was additionally adjusted for albuminuria and intake of mono- and disaccharides.

To identify effect modifiers, multiple interaction terms with sex were included in the model. In case of a significant interaction term, we performed a stratified analysis using the fully adjusted model. Additionally, we calculated the percentage difference (95% CI) in calprotectin levels per 1 SD higher or compared to reference category or correlate.

A logistic regression was performed to assess the potential role for calprotectin in the development of CVD, compared with other inflammation parameters (CRP, leukocytes, thrombocytes, serum albumin). To increase comparability among parameters, Z-scores were calculated and used in the logistic regression analysis, adjusted for the determinants found in the linear regression analysis. Nutritional intakes were adjusted for total energy intake by the residual method [33]. Therefore, we used the mean total caloric intake of our population in the regression equations.

Sensitivity analysis was performed in which we repeated analyses after addition of polysaccharides, fatty acids and dietary protein as confounders in the fully adjusted model. Additional analyses were performed with inclusion of HbA1c (mmol/mol), insulin use (*n*, %) and diabetes duration (years) to the multivariate logistic regression model for CVD.

## 5. Conclusions

Higher serum calprotectin levels are associated with smoking status, a well-known direct cause of inflammation of the arteria, with the intake of mono- and disaccharides and with albuminuria in patients with T2DM. It is plausible that serum calprotectin is an inflammation marker since it is associated with CVD. These first results in T2DM patients appear to be a good starting point to investigate the potential role of serum calprotectin as a biomarker in prospective studies.

## Figures and Tables

**Table 1:** Univariate correlation coefficients between determinants and log serum calprotectin levels.

Variable	Total Population	R	*p*-Value
N	362		
Serum calprotectin (mg/L)	1.04 [0.73–1.46]		
Age (years)	63 ± 9	−0.035	0.51
Body Mass Index (kg/m^2^)	32.7 ± 5.8	−0.025	0.65
Normal weight (BMI < 25 kg/m^2^)	18 (5)	Ref	
Overweight (BMI 25–30 kg/m^2^)	110 (30)	0.060	0.61
Obese (BMI > 30 kg/m^2^)	234 (65)	0.061	0.60
Urinary creatinine excretion (mmol/24 h)	13.8 ± 4.9	−0.031	0.62
Waist circumference (cm) ^1^	112 ± 13	0.030	0.57
Smoking status			
Never smoker	110 (30)	Ref	
Former smoker	190 (53)	0.135	0.022
Current smoker	62 (17)	0.266	<0.001
Alcohol consumption			
No alcohol consumption	130 (36)	Ref	
<1 consumption per day	140 (39)	−0.031	0.61
>1 consumption per day	92 (25)	0.076	0.23
Physical activity ^1^	205 (57)	0.066	0.21
Systolic blood pressure (mmHg)	139 ± 16	−0.070	0.18
Diastolic blood pressure (mmHg)	76 ± 10	−0.091	0.08
HbA1c	57 ± 12	−0.063	0.23
Insulin use (*n*, %)	227 (63)	−0.016	0.77
Diabetes duration (years)	11 [7–18]	−0.015	0.78
eGFR (mL/min/1.73 m^2^)	78 ± 24	−0.087	0.10
Albuminuria (*n*, %)	110 (30)	0.135	0.011
**Nutrition**			
Energy (kcal/day)	1993 ± 640	0.048	0.40
Protein (g/day)	78 ± 12	−0.022	0.68
Vegetable protein (g/day)	27 ± 6	−0.084	0.11
Animal protein (g/day)	51 ± 13	0.015	0.78
Fatty acids (g/day)	88 ± 14	0.015	0.78
Carbohydrates (g/day)	205 ± 34	0.056	0.29
Mono- and disaccharides (g/day)	91 ± 28	0.127	0.015
Polysaccharides (g/day)	111 ± 26	−0.063	0.23
Fibre (g/day)	20 ± 5	−0.011	0.83
Estimated sodium intake (mg/day) ^2^	4284 ± 1838	−0.055	0.32

Values are given as mean ± standard deviation, median [interquartile range] or number (percentage). Sex-adjusted standardized linear regression coefficients were estimated to assess the cross-sectional correlations between various determinants and log calprotectin levels. ^1^ Missing values for waist circumference (*n* = 3), physical activity (*n* = 9) and HbA1c (*n* = 1) ^2^ Based on 24-h urinary sodium excretion.

**Table 2:** Multivariate linear regression analysis regarding increased serum calprotectin levels in 362 patients with T2DM in the DIALECT-1 population.

	Model 1		Model 2		Model 3	
	B	*p*-Value	B	*p*-Value	B	*p*-Value
Age	−0.035	0.51	−0.019	0.72	−0.036	0.48
Former smokers			0.137	0.021	0.133	0.023
Current smokers			0.264	<0.001	0.269	<0.001
Mono- and disaccharides (g/day)					0.143	0.005
Albuminuria					0.132	0.011

Forward multivariate linear regression analysis with log calprotectin as dependent variable (standardized beta, *p*-value). Model 1 is adjusted for sex and age, model 2 is adjusted for sex, age and smoking status and model 3 is additionally adjusted for albuminuria and intake of mono- and disaccharides.

**Table 3:** Multivariate linear regression analysis with percentage difference (95% CI) in calprotectin levels per 1 SD higher or compared to reference category or correlate in 362 patients with T2DM in the DIALECT-1 population.

Variable	Standardized Beta	*p*-Value	Percentage Difference (95% CI)
Sex	0.155	0.003	20 (6 to 35)
Former smokers	0.133	0.023	16 (2 to 33)
Current smokers	0.269	<0.001	51 (27 to 79)
Intake of mono- and disaccharides	0.143	0.005	0.2 (0 to 0.5)
Albuminuria	0.132	0.011	18 (4 to 34)

Linear regression model with log calprotectin level as dependent variable. Reference category for smoking are never smokers.

**Table 4:** Serum calprotectin as predictor for cardiovascular disease (CVD), compared with other inflammatory parameters in 362 patients with T2DM in the DIALECT-1 population.

Variable	Beta (95% CI)	*p*-Value
ZCalprotectin	1.356 (1.037–1.772)	0.026
ZCRP	1.093 (0.877–1.363)	0.43
ZLeukocytes	1.248 (0.972–1.604)	0.08
ZThrombocytes	1.026 (0.811–1.299)	0.83
ZSerum albumin	0.942 (0.740–1.200)	0.63

Logistic regression model with CVD as study outcome and Z-scores for inflammation parameters. Adjusted for age, sex, smoking status, intake of mono- and disaccharides and albuminuria.

## Abbreviations

T2DM	Type 2 Diabetes Mellitus
CVD	Cardiovascular diseases
CRP	C-reactive protein
